# Benign metastasizing leiomyoma presenting as multiple cystic pulmonary nodules: a case report

**DOI:** 10.1186/s12905-017-0435-6

**Published:** 2017-09-12

**Authors:** Yeong Hun Choe, So Yeon Jeon, Yoon Chae Lee, Myung Ja Chung, Seung Yong Park, Yong Chul Lee, So Ri Kim

**Affiliations:** 1 Department of Internal Medicine, Chonbuk National University Medical School, San 2–20, Geumam-dong, Deokjin-gu, Jeonju, Jeonbuk 561-180 South Korea; 2 Department of Pathology, Chonbuk National University Medical School, Jeonju, South Korea; 3 Research Institute of Clinical Medicine of Chonbuk National University-Biomedical Research Institute of Chonbuk National University Hospital, Jeonju, South Korea

**Keywords:** Benign metastatic leiomyoma, Lung, Multiple cysts

## Abstract

**Background:**

Benign metastatic leiomyoma (BML) is an extremely rare disease. Although uterine leiomyomas are benign histologically, they can metastasize to distant sites. While the incidence is very low, the lung is the organ most frequently affected by BML. Pulmonary BML usually presents as numerous well-defined nodules of various sizes, while the cavitary or cystic features in the nodules are rarely observed on radiologic images.

**Case presentation:**

A 52-year-old woman complained of cough and dyspnea for one month. She had been previously diagnosed with uterine leiomyoma and had undergone total hysterectomy about 14 years prior. High-resolution computed tomography (CT) images showed that there were multiple cystic nodules of various sizes in both lungs. Pathologic examination revealed that the pulmonary nodule had complex branching glandular structures lined by a single layer of simple cuboidal to columnar epithelium that was surrounded by abundant spindle cells. Additional immunohistochemistry data suggested that pulmonary nodule diagnosis was BML-associated uterine leiomyoma.

**Conclusion:**

In this report, we introduce an interesting case of pulmonary BML that presented as a combination of various kinds of nodules including simple round nodules, simple cysts, and cysts with a solid portion, which are very rare radiologic features of BML in lung. In addition, when the patient is a woman of reproductive age, physicians should meticulously review the gynecological history and suspect BML when there are various cystic pulmonary lesions.

## Background

Benign metastasizing leiomyoma (BML) is a very rare condition that has been reported in association with uterine leiomyoma [1]. BML usually affects women of reproductive age with a history of uterine myoma. Although uterine leiomyomas are histologically benign, they can metastasize to distant sites such as lung, skin, bone, mediastinum, lymph node, muscular tissue, heart, and retroperitoneum [2, 3]. Among them, lung is the organ most affected.

Patients with pulmonary BML are usually asymptomatic. Therefore, the lesions are frequently detected on radiologic examination during medical check-up. Common radiographic features of pulmonary BML are multiple pulmonary solid nodules of various sizes [4–9]. However, there are few reports of BML presenting as cystic or cavitary features.

Herein, we describe a case of pulmonary BML exhibiting an intriguing radiologic finding on chest computed tomography (CT), a combination of various nodules having a cystic nature as well as mixed solid and cystic features.

## Case presentation

A 52-year-old woman complained of cough and dyspnea for one month. There was no history of weight loss, hemoptysis, chest pain, smoking, or environmental and/or drug exposure. Physical and laboratory examination including spirometric analysis did not show any relevant abnormality. About 14 years prior, the patient was diagnosed with uterine leiomyoma and had undergone total hysterectomy.

**Fig. 1:**
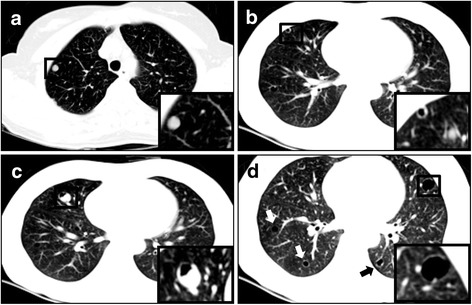
Chest CT reveals multiple cystic nodules. **a** A 6 mm-sized round nodule in the right upper apical segment. **b** A 1 cm-sized cystic lesion with a thin wall in both lungs’ parenchyma. **c** A 1 cm-sized cystic and well demarcated lesion with solid portion in the right middle lobe. **d** Multiple cystic nodules (arrows) with various sizes are in both lungs. CT: Computed tomography

**Fig. 2:**
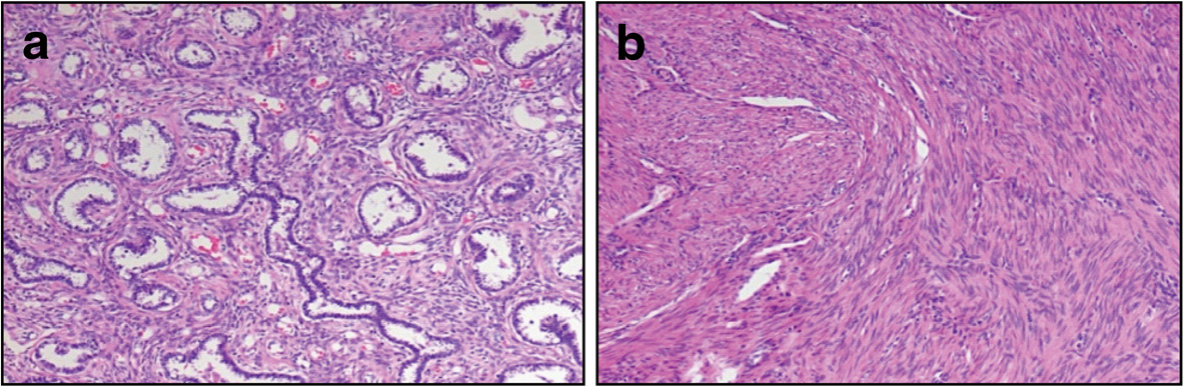
Representative H&E-stained sections of the pulmonary nodule (**a**) and the uterine mass (**b**). **a** The pulmonary nodule showed complex branching glandular structures lined by a single layer of simple cuboidal to columnar epithelium surrounded by abundant spindle cells. There was no atypia or mitotic activity in either the epithelial or the spindle cell components (H&E staining, × 100). **b** The uterine mass resected 14 years prior consisted of myometrium-derived smooth muscle cells and extracellular matrix

**Fig. 3:**
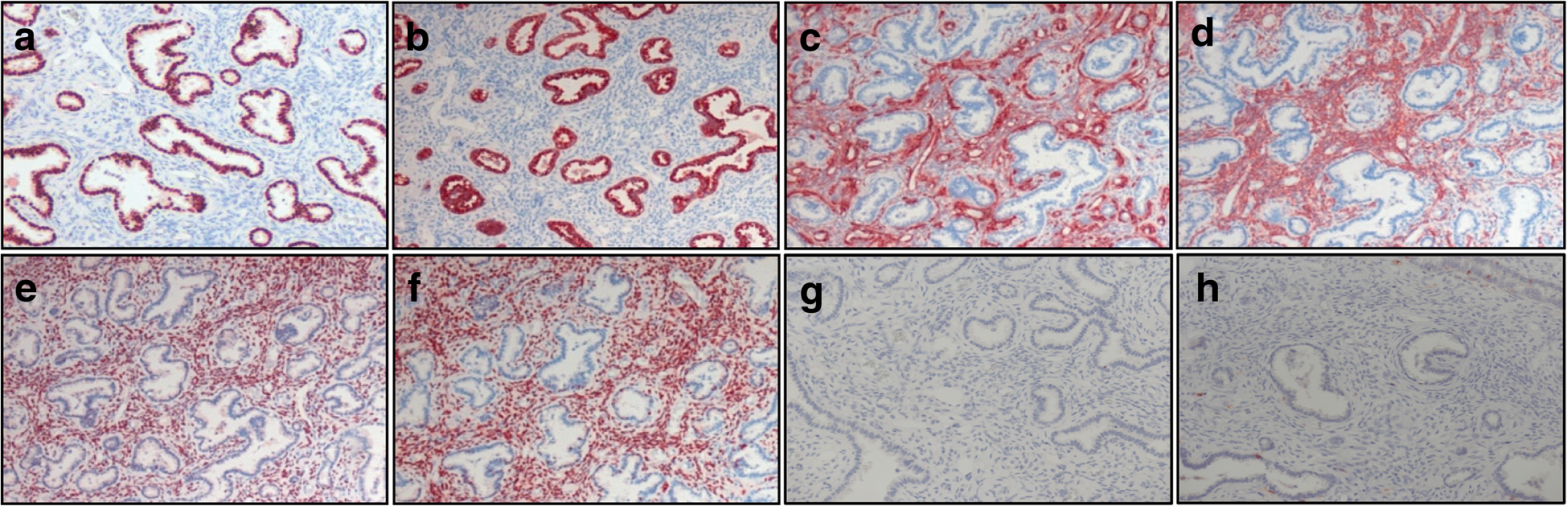
Immuno-histochemical staining sections of the pulmonary nodule. Positive immunoreactivity for TTF-1 (**a**), EMA (**b**), SMA (**c**), desmin (**d**), ER (**e**), PR (**f**), HMB45 (**g**), and S100 (**h**) was observed. TTF-1: Thyroid transcription factor-1, EMA: Epithelial membrane antigen, SMA: Smooth muscle actin, ER: Estrogen receptor, PR: Progesterone receptor

Chest X-ray showed no definite lesions in either of the lung fields. However, high resolution CT showed multiple cystic nodules of variable size (1.0 cm to 1.4 cm in diameter) in both lungs (Fig. 1). Some nodules were mixed with solid portions, but pure solid nodules were also observed. After reviewing the chest imaging, we performed several diagnostic tests to define possible etiologies for these multiple cystic lesions, including infection, malignancy, and immunologic diseases. There was no definitive endobronchial lesion on bronchoscopic examination, and bronchial washing cytology was negative for malignancy. Bronchoalveolar lavage cultures were negative for bacteria, mycobacterium, virus, and fungi. For pathologic diagnosis, we performed wedge resection of the pulmonary nodule in the right middle lobe. The surgical specimen contained a well-demarcated cystic nodule possessing a pale yellow-colored solid portion. Grossly, the nature of the nodule was firm with a size of 1.2 cm × 1.0 cm in diameter. Microscopic findings showed complex branching glandular structures lined by a single layer of simple cuboidal to columnar epithelium were surrounded by abundant spindle cells. There was no atypia or mitotic activity in either the epithelial or the spindle cell components (Fig. 2). Immunohistochemically, the epithelial lining cells were positively stained for epithelial membrane antigen (EMA) and thyroid transcription factor (TTF)-1. In addition, the tumor cells contained smooth muscle actin (SMA). Desmin, estrogen receptor (ER), and progesterone receptor (PR) were also found in the cells of the nodule, while tumor cells were negative for HMB-45 and S100 (Fig. 3). Based on these pathologic features, we diagnosed the pulmonary nodules as BML associated with uterine leiomyoma. We decided to monitor the patient’s pulmonary lesions without further treatment. She had no aggravation of BML for 6 years since diagnosis.

## Discussion

Pulmonary BML was first identified by Steiner in 1939 [1], and as he believed it to be a primary lung neoplasm, BML was first referred to as fibroleiomyomatous hamartoma. BML is a rare pathologic condition and difficult to diagnose mainly due to the lack of clinical suspicion. BML occurs in young premenopausal women aged 35 to 55 years with a history of uterine myoma or surgery such as hysterectomy or myomectomy. Although about 130 BML cases have been reported previously [2, 10–14], cystic pulmonary BML has not been reported. Based on information from previous cases, the mean interval between hysterectomy and the development of BML is 14.9 years. Most of the patients have favorable outcomes with a calculated median survival of 94 months [3].

Typical radiologic findings of pulmonary BML are numerous well-defined solid pulmonary nodules sized from a few millimeters to several centimeters, whereas there are few reports of cystic or cavitary nodular manifestation [4–9]. We previously reported that the typical radiologic feature was a round shaped solid pulmonary nodule on chest CT [10]. To our knowledge, simultaneous presentation of various radiologic features of pulmonary BML nodules like this current case has not been reported.

In clinical practice, physicians should carefully consider the following etiologies for multiple cystic lesions: neoplastic origin, infections by bacteria, fungi, and/or parasites, immunologic diseases, pulmonary embolism, pneumoconiosis, localized bronchiectasis, pulmonary lymphangioleiomyomatosis (LAM), pulmonary Langerhans cell histiocytosis (PLCH), and so on, since there are many more common causes than pulmonary BML. Moreover, solid contents within a cavity may be a clue that the cystic lesions were caused by infectious processes such as invasive aspergillosis or by necrotic cancer [15]. In this present case, we decided to confirm the etiology of nodular lesions pathologically, considering these differential diagnoses. The site of the target nodule on the right middle lobe was suitable for wedge resection to obtain adequate tissues for diagnosis.

Pathologically, BML exhibits extrauterine proliferations of bland smooth muscle cells and mitotic inactivity [16, 17]. There is no evidence of nuclear pleomorphism or necrosis in BML. BML usually shows a similar histologic appearance to that of a primary benign uterine tumor, i.e., leiomyoma. Therefore, immunohistochemically positive staining for ER and/or PR provides the diagnostic keys for BML. Exclusion of LAM is important in the diagnosis of cystic BML because it is a representative lung disorder that presents with multiple pulmonary cavities in middle-aged women. Both BML and LAM show some similarities; smooth muscle cell proliferation and expression of ER and PR in tumor cells [18]. However, pathologic patterns and immunochemical properties are helpful to distinguish BML from LAM. Smooth muscle cells proliferate with a peribronchial pattern in BML, while LAM shows proliferation of atypical smooth muscle cells along with lymphatics, blood vessels, and small airways. Moreover, the positivity of HMB-45 in the tumor cells is more important than anything else [19]. In the current case, the tumor was composed of abundant spindle cells that showed positivity for SMA, desmin, ER, and PR and negativity for HMB-45 and S100, suggesting a pathologic diagnosis of BML.

Some studies have shown cytogenetic abnormalities in BML [20]. If BML is hypothesized to arise from uterine myoma, demonstration of shared genetic abnormalities between the primary and metastatic lesions may provide valuable information for a diagnosis of BML, especially when morphological and histopathologic features are unusual. Examples of proposed abnormalities include 19q and 22q terminal deletion, X-chromosome inactivation, and 12q15 rearrangements involving the HMGA2 [20, 21]. These gene analyses appear to be helpful for the correct diagnosis of BML and differential diagnosis.

To date, no standard treatment for BML has been introduced. Current therapeutic options include careful observation, surgical resection of pulmonary nodules, bilateral debulking oophorectomy, hormonal therapy, and radiation therapy to the ovary and chemotherapy [11]. In our patient, there was no mitotic figure in the tumor. Thus, we decided careful observation for pulmonary BML was critical.

## Conclusions

In this report, we introduced an interesting case of pulmonary BML that manifested as multiple nodules with various radiologic features including cystic, mixed typed and solid natures. This radiologic presentation suggests that physicians should include pulmonary BML as a differential diagnosis when they encounter multiple pulmonary cystic nodules with diverse characteristics, especially in the case of middle-aged women with a history of uterine pathology.

## Abbreviations

BML: Benign metastasizing leiomyoma
CT: Computed tomography
EMA: Epithelial membrane antigen
ER: Estrogen receptor
LAM: Lymphangioleiomyomatosis
PLCH: Pulmonary Langerhans cell histiocytosis
PR: Progesterone receptor
SMA: Smooth muscle actin
TTF-1: Thyroid transcription factor-1
